# Metagenomic Data Utilization and Analysis (MEDUSA) and Construction of a Global Gut Microbial Gene Catalogue

**DOI:** 10.1371/journal.pcbi.1003706

**Published:** 2014-07-10

**Authors:** Fredrik H. Karlsson, Intawat Nookaew, Jens Nielsen

**Affiliations:** 1 Department of Chemical and Biological Engineering, Chalmers University of Technology, Gothenburg, Sweden; The Pennsylvania State University, United States of America

## Abstract

Metagenomic sequencing has contributed important new knowledge about the microbes that live in a symbiotic relationship with humans. With modern sequencing technology it is possible to generate large numbers of sequencing reads from a metagenome but analysis of the data is challenging. Here we present the bioinformatics pipeline MEDUSA that facilitates analysis of metagenomic reads at the gene and taxonomic level. We also constructed a global human gut microbial gene catalogue by combining data from 4 studies spanning 3 continents. Using MEDUSA we mapped 782 gut metagenomes to the global gene catalogue and a catalogue of sequenced microbial species. Hereby we find that all studies share about half a million genes and that on average 300 000 genes are shared by half the studied subjects. The gene richness is higher in the European studies compared to Chinese and American and this is also reflected in the species richness. Even though it is possible to identify common species and a core set of genes, we find that there are large variations in abundance of species and genes.

## Author Summary

Our bodies are home to a myriad of microbial cells and our intestinal tract is especially densely populated with bacteria. Alterations in the composition of the gut microbiota have been associated with common human diseases. By sequencing the genomes of the microbes, the metagenome, detailed information about who is there and their capabilities can be obtained. In this paper, a method for analyzing metagenomic data is presented together with an analysis of gut metagenomes from 4 different studies and 3 different continents. We identify a core set of genes and species were identified but the abundance of core components differs between study populations. A catalogue of gut microbial genes from the 4 studies was constructed containing more than 11 million genes.

## Introduction

Metagenomic sequencing of the human microbiome has contributed to our understanding of the microbial communities that live in symbiosis with humans and their genomic capabilities [1], [2]. The human gut microbiome is associated with a range of metabolic diseases and likely influences our physiology and nutrition [3], [4], [5], [6]. To discern the associations between the gut microbiome and human health, metagenomic sequencing by generating millions of short reads from community genomes is a very powerful tool that generates vast amounts of information about the microbiome. To analyze the functional content of a metagenomic data set, its diversity and content, bioinformatics tools together with computational resources are necessary. By aligning the reads to a database of reference genomes or genes assembled *de novo* from the reads themselves and counting the reads on each reference sequence, a quantitative measure of the microbiome composition can be obtained. The analysis also involves preprocessing such as quality assessment and filtering out human reads.

Several methods exist for either performing de novo assembly of the metagenomic data to predict gene sequences from longer contigs such as SOAPdenovo [7], velvet [8] and MOCAT [9] which is a dedicated pipeline for metagenomic *de novo* assembly. The *de novo* assembly tools are important because the available genomic databases do not yet include complete genomes for many organisms present in metagenomic samples. Tools for taxonomic assignment of metagenomic reads have been developed and these include Phylophytia [10], PhymmBL [11] and MetaPhlAn [12]. These tools rely on a database of reference genomes that is either used for training a classifying model or for direct alignment of sequence reads.

To address the problem of quantitative characterization of a metagenome data set, we have developed a tool for quality control, filtering reads and counting alignments to reference genomes and a gene catalogue database in one step. Furthermore, downstream tasks such as handling a large number of samples and annotating the alignment counts to taxonomic and functional databases are handled. Handling an abundance table of several hundred samples and millions of gene features puts special requirements on efficient implementation. This requires a machine with a large amount of RAM and efficient data management codes. We have tested MEDUSA on four gut metagenomic datasets from three continents and evaluate its performance by mapping to two databases, one reference genome catalogue made up of 1747 bacterial and archaeal genomes and a gene catalogue constructed in this study.

One important question in the field of the human gut microbiome is whether there is a common core of species and genes and how variable the microbiome is between different individuals. A core of gene functions was identified in an American population of 18 individuals but using 16S rRNA sequencing on 154 individuals did not identify a core at the species level [13]. By using metagenomic sequencing on 124 individuals from Denmark and Spain, a species core was identified and as well a core of almost 300 000 genes was identified in at least half the population [2]. An unanswered question is whether there is a core microbiome across continents. Is there a core at the species level and at the gene level? To address these questions we used the data from four studies and found core species and genes. The core genes are also the most abundant genes but each individual also carries a large number of genes that are not shared with a majority of the population or are unique. Interestingly we found that the abundance of core species varies substantially between the studies.

## Results

### MEDUSA overview and design principle

**Figure 1:**
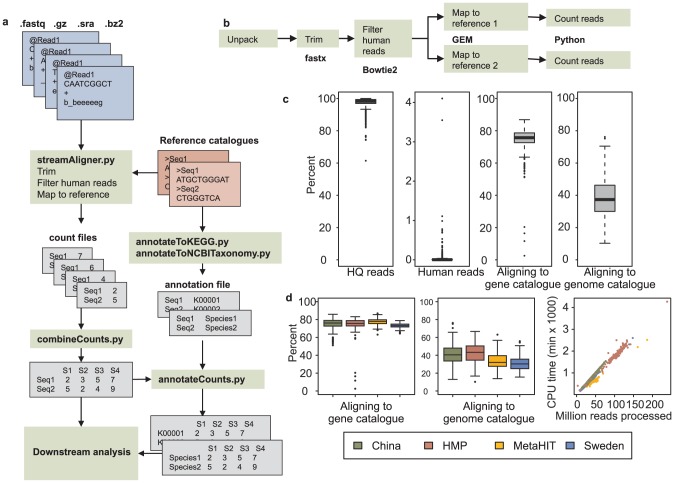
The MEDUSA pipeline and its application to 4 gut metagenome datasets. (a) An overview of the MEDUSA pipeline and its functions is shown. Input data is fastq and can be compressed in various ways. MEDUSA counts reads aligning to a reference catalogue and outputs count files that can be annotated and analyzed. (b) The alignment function is implemented using linux pipes which reduces file IO substantially and integrates the quality control, filtering and aligning to a database into one step. (c) Data statistics of the human gut samples analyzed in this study. Most reads (>90%) pass the quality control step and few samples have any substantial contamination of human DNA. Overall, the reads align to the gene catalogue to a larger extent compared to the genome catalogue. (d) Percent of reads aligning to the gene and genome catalogues are shown for each study. Furthermore, for each sequencing run, the processing time and the number of reads are shown and scales linearly.

MEDUSA is an integrated pipeline for analysis of short metagenomic reads, it contains modules for mapping reads to reference databases, combining output from several sequencing runs and manipulating the tables of read counts and testing for differential abundance (Figure 1a). Python was used for creating a pipe to stream metagenomic reads stored in fastq files (can be compressed with gz, bzip2 or in SRA archives) through a quality control step, filtering out human reads and mapping reads to two databases simultaneously, without the need for writing intermediate files (Figure 1b). By streaming reads in a pipe, time consuming disk IO is eliminated and disk space is saved. MEDUSA also contains tool for combining and analyzing a table of counts in numpy which facilitates a fast framework for manipulating a table that had several hundred by several millions entries. These tools include performing rarefaction to sample the reads to the same depth of sequencing, testing for differential relative abundance and plot relative abundance for selected features. The reference catalogues used can be a gene catalogue and a genome catalogue and this approach has been used previously [2], [3]. MEDUSA can merge count tables of genes and genomes with annotation information to generate a KEGG ortholog abundance and taxonomic table.

### Species catalogue construction

In this study, four of the largest published gut metagenomic datasets to date were included and compared. The subjects are from United States of America (Human microbiome project, HMP) [1], China [4], Denmark, Spain (MetaHIT) [2] and Sweden [3], all together containing 40 billion metagenomic reads and 782 samples. All samples were sequenced on the Illumina platform with read lengths from 44 to 100 base pairs.

A non-redundant catalogue of species genomes was constructed based on the results of a method using 40 universal single copy phylogenetic marker genes used for clustering prokaryotic genomes into species [14]. The catalogue contains 1747 species genomes downloaded from NCBI Genbank and the full list of genomes is presented in Table S1. The quality controlled and filtered reads were aligned to the genome catalogue and the number of aligning reads to each contig in the database was counted.

### Data mapping

Reads files from the four studies were used as input to the function *streamAligner*. This function can take a number of compressed fastq files as input and will produce a count file for each input file and reference database. The function produces a log file for each input file with mapping statistics and output from the various software used in the stream such as fastx and Bowtie2. The function *streamAligner* can easily be parallelized by starting many instances of the function; each instance will look in the list of files supplied and start working on unprocessed files given that all instances have access to the same file system. The input number of reads for each study were on average 40±12, 102±28, 45±18 and 31±18 million single end reads per sample for the studies China, HMP, MetaHIT and Sweden, respectively. Most of the sequencing runs have a high quality with almost 98% of the reads passing the quality cutoff (Figure 1c, Table S2). Out of the high quality reads, on average only 0.023% aligned to the human genome although the HMP data had been cleaned for human reads before submission to a public database. It is worth to note that the degree of human reads in a sample is highly variable with a few samples with considerable fraction of human reads and therefore the filtering of human reads is important even in gut metagenome datasets where the fraction of human reads is low compared to data from other body sites [1]. Out of the HQ non-human reads, 75% could align to the gene catalogue while 39% could be aligned to the genome catalogue which is similar to previous results or alignment to gene and genome catalogues [2], [3]. This indicates that there are still species in the gut that have not yet been identified. The function *combineCounts* takes a range of input files and a file mapping sequence runs to a sample since some samples could be sequenced in several runs. The output of *combineCounts* is a large abundance matrix which has aligned features as rows and samples as columns.

We compared our results of the genus abundance to another tool, Metaphlan [12] which uses clade specific marker genes from reference genomes for taxonomic profiling of metagenomes. HMP samples profiled with Metaphlan were compared to the results using MEDUSA on the genus level and the comparison accounts on average for 99.5±0.46% and 98.1±2.1% of the reads aligned reads, respectively. Comparing the 137 samples that were shared, we find that the Pearson correlation between the profiles are 0.95±0.06 (Table S3), indicating that the two methods produce very similar results. Performance of Metaphlan has been reported to be 450 reads per second on a single CPU [12]. MEDUSA was here performing with a throughput of 938 reads per second (AMD Opteron 6220), but then quality control, human filtering and alignment to the reference genomes and gene catalogue were done simultaneously.

**Figure 2:**
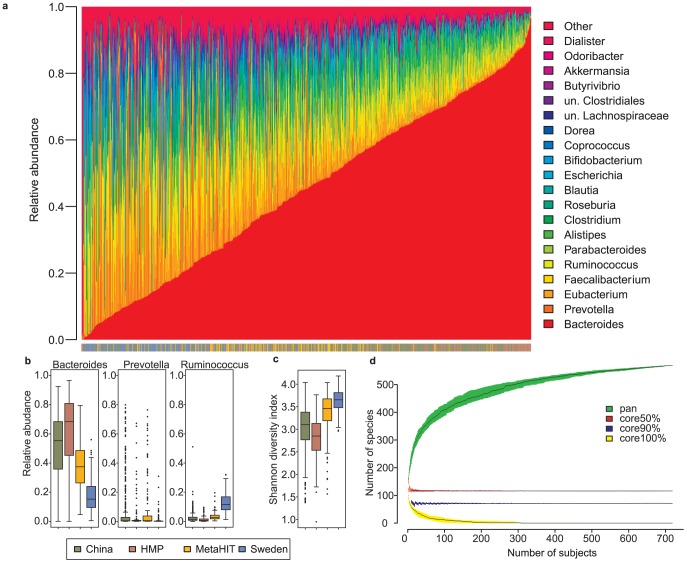
Taxonomic analysis of the gut metagenome. (a) Genus abundance of each sample ordered by increasing *Bacteroides* relative abundance. There is a continuous gradient of increasing *Bacteroides* relative abundance in the studied samples. The 20 most abundant genera are shown, whereas the rest of the annotated reads are grouped into other. (b) Boxplots showing the relative abundance of *Bacteroides*, *Prevotella* and *Ruminococcus*. The *Prevotella* abundance is low in most samples but a few samples have a major *Prevotella* abundance. (c) Shannon diversity index of the species abundance shows that Swedish and Metahit samples have a higher diversity compared to Chinese and American. (d) Pan and core species with a relative abundance above 10^−4^ in the subjects (repeated samples from the same subject excluded). The core percentage means that a species was present in at least % of the subjects.

The taxonomic profiles at the species and genus level of all samples were determined by analyzing the aligned reads to reference genomes. The most abundant genus in the cohort was *Bacteroides* but the inter-individual variation was large spanning from almost 1 to 0 (Figure 2a), the top 20 most abundant genera account for 93±8% of the annotated reads. The most abundant species were from *Bacteroides*, *Faecalibacterium* and *Eubacterium* with inter-individual variations in abundance spanning several orders of magnitude (Figure S1). The abundance of *Bacteroides* was higher in HMP and Chinese samples compared to Metahit and Swedish samples and the latter had higher abundance of *Ruminococcus* (Figure 2b). The abundance of other genera also varied across study populations and in general the Swedish and to some extent the Metahit population had more Firmicutes, e.g. *Faecalibacterium*, *Eubacterium*, *Clostridium* and *Dorea* (Figure S2). Analyzing the diversity of the species found in the samples shows that the diversity is highest in the Swedish samples followed by MetaHIT which are also less dominated by *Bacteroides*. Heatmaps of species and genera abundance are shown together with a clustering of samples in Figure S3 and S4. Using the species abundance profiles to calculate the diversity of species shows that MetaHIT and Swedish samples have a higher diversity compared to American and Chinese. The higher diversity in these samples is likely due to a smaller dominance by *Bacteroides* which is not replaced by one species or genera but several different Firmicutes species.

To address whether there is a core of species that is shared by subjects from the different cohorts, we looked at species with a relative abundance above 0.0001 across subjects and found 116 species above this threshold in 50% of the subjects and 71 species above the threshold in 90% of the subjects (Figure 2d and Table S4). This indicates that there is a common core of species shared across all cohorts but their abundance differs extensively. Since the size of the species core have been shown to be affected by the depth of the analysis using the HITChip [15] we investigated the sensitivity using metagenomic sequencing. The performed analysis shows that the size of the core is relatively insensitive to the cutoff used for abundance (Figure S5).

Three enterotypes or clusters of stratified intestinal microbiota composition were suggested [16] and here we investigate the existence of enterotypes in the combined cohorts. The strongest support was found for three clusters with an average Silhouette width of 0.29 (Figure S6). The driver genera were *Bacteroides*, *Prevotella* and *Ruminococcus* as originally proposed (Figure S7). However, the three enterotypes were strongly associated with the 4 study cohorts, China and HMP samples were enriched in enterotype 1, Metahit evenly distributed among the three and Sweden enriched in enterotype 3 (Table S5 and Figure 2b). When studying only the Danish samples from the Metahit cohort and comparing to the outcome in the original population, there is a 96% agreement between the clustering results (Table S6). Ranking the subjects according to their relative abundance of *Bacteroides* indicates that there is a smooth gradient but *Prevotella* shows a bimodal distribution indicating that subjects fall into primarily two categories with the abundance either being >10% or <1% (Figure S8).

### Gene catalogue construction

**Figure 3:**
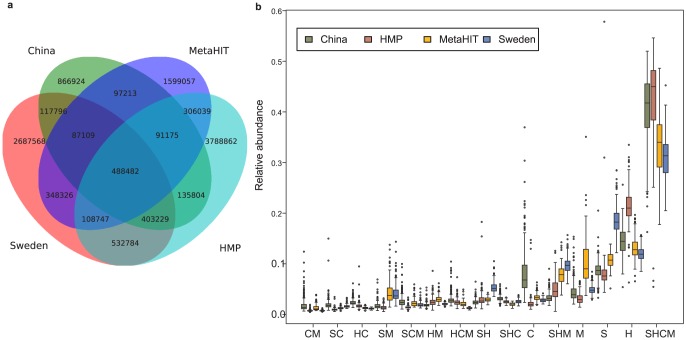
Gene catalogue construction and abundance. (a) The Venn diagram shows how the 11 659 115 genes were shared in the 4 studies based on the merge of the 4 non-redundant gene catalogues. A core of 488 482 genes were found in all studies whereas a large part of the genes were unique to each study. (b) Relative abundance of genes grouped into how they are shared in the Venn diagram. The shared genes are also the most abundant genes followed by the unique genes to each study. Each field in the Venn diagram is denoted by the first letter of the study.

We extended the human gut microbial gene catalogue by merging data from the four different gut metagenome studies. Contigs from each study were downloaded and genes were predicted, in total 72.5 million genes were predicted. 67 million genes were predicted from the individual assemblies of samples and 5.5 million genes were predicted from the global assemblies that were performed on unassembled reads (Figure S9). Genes from each individual study were then clustered based on their sequence similarity using Uclust [17] and a 95% identity and 90% coverage cutoff. In a final step, the NR genes from each study were then clustered using the same criteria as above and a global human gut microbial gene catalogue was obtained containing 11 million genes. Each study showed a substantial number of unique genes while the common genes to all studies was 488 482 and 2.7 million genes were shared between any two studies whereas almost 9 million genes were unique to a single study (Figure 3). The largest number of unique genes was found in the HMP samples and these were also the deepest sequenced. The lowest number of unique genes was found in the Chinese cohort on which a global assembly of unassembled reads from individual assemblies was not done. The largest overlap between two studies was found between the Swedish and HMP studies with over 1.5 million shared genes.

Although each study contained many unique genes from *de novo* assembly, we wanted to study the abundance of the shared and unique genes in each subject. To get a quantitative measure of gene abundance, reads were mapped back to the gene catalogue as described above and in Methods. On average 38±8% of reads in each sample mapped to the core genes (488 482) found in all studies (Figure 3). A similarly large part of reads mapped to study-unique genes (36±4%). This indicates that there is a substantial part of the microbiome that is shared but also that low abundant genes are unique to individuals. If the abundance is also normalized to the number of genes in each category it is clear that the most abundant genes are shared (Figure S10).

**Figure 4:**
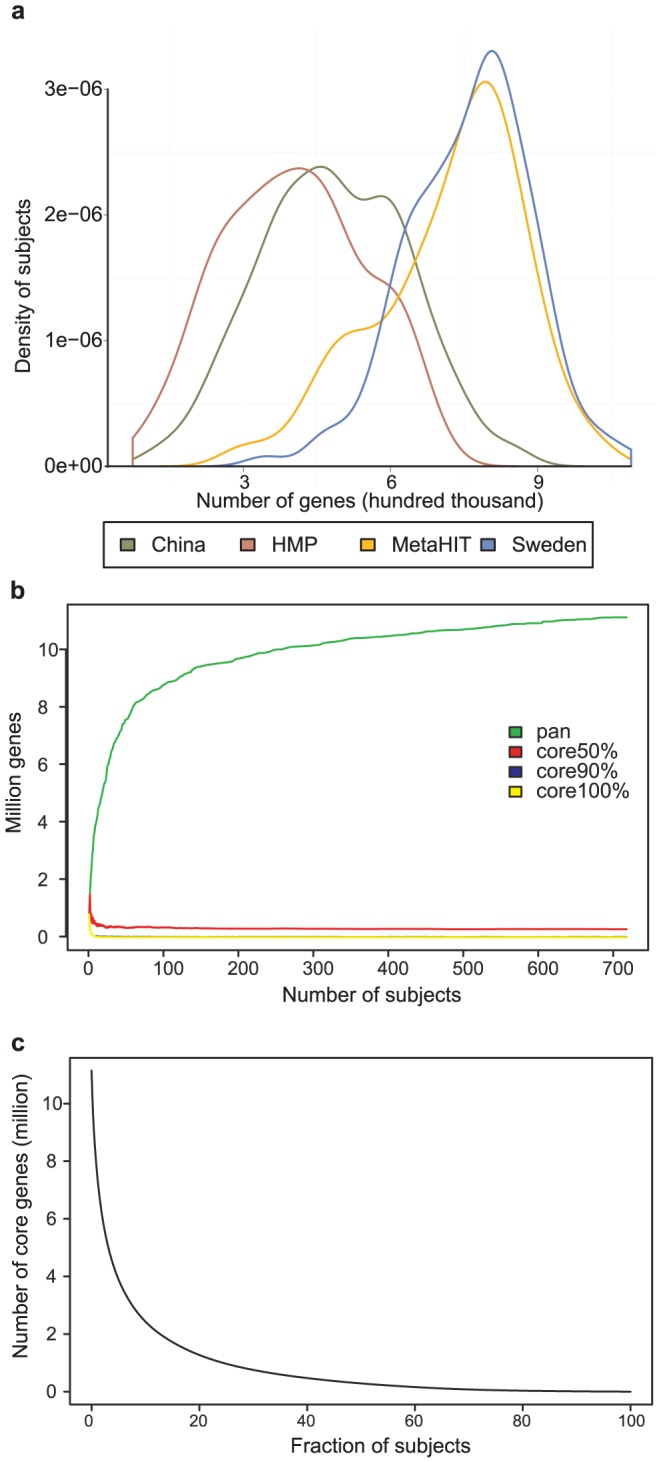
Gene richness and pan and core genes. (a) Number of genes in each sample using 11 million reads is shown as a smoothed histogram. European samples have a higher gene richness compared to the Chinese and American. (b) The number of genes as a function of the number of samples. The definitions of the cores are the same as in Figure 2. The size of the core50% is 283 705 genes. (c) Shows the number of core genes as a function of the inclusion criteria (% of the population having the gene).

To determine the richness of the microbiota using the gene catalogue, aligned reads were counted and two reads were required to call a gene present in a sample. Comparison of the gene richness in the 4 studies shows that the European samples have a higher gene count compared to Chinese and HMP samples (Figure 4a). When counting genes, all samples were rarefied to the same number of reads, 11 million, in order to remove the effect of different sequencing depth and 23 samples were removed because of limited sampling depth. Regardless of rarefaction, European samples showed a higher gene richness compared to Chinese and HMP samples. Recently the gene richness has been associated with lower BMI and favorable metabolic markers in a study of Danish subjects [6]. All HMP subjects are reported to be healthy but still show a markedly lower gene richness compared to the two European cohorts. Since the gene richness is so closely associated with the different studies, we did not investigate any associations between gene richness and health status, as methodological differences cannot be ruled out. In a study of American twins, the association between gut microbiota richness and obesity has also been reported previously using 16S rRNA sequencing [13]. Low diversity of the microbiota has been reported to be associated with inflammatory bowel disease [18] and inflammation in elderly [19]. A comparison of the diversity between populations also found that American subjects had a less diverse microbiota compared to Amerindians from Venezuela and Malawians [20]. The differences became evident after 3 years of age, but not in younger subjects.

Despite differences in diversity, there is a core of genes found in a majority of the subjects. By counting the genes present in at least 50% of the population we found 283 705 genes which indicated that a large portion of the genes carried by an individual is shared. In the original MetaHIT study of 124 subjects, each individual carried just above 536 112 genes on average [2]. A core of genes was identified of 294 110 genes being present in at least half the MetaHIT population which also means that a large number of genes were only found in one or a few subjects. However, there are only 3 genes shared by all subjects of this study (Figure 4b,c). The number of genes shared by at least 50% of the subject is stable when more subjects are added and it can therefore be expected that this number will be stable also when more subjects are included. However, the number of core genes is highly dependent on the fraction of subjects required to carry the gene (Figure 4c) e.g. there are 1.3 million genes shared by at least 20% of the population. The pan genome is quickly increasing by the number of subjects which also means that most genes are shared by at least 2 individuals and in fact over 10 million genes are found in at least 2 individuals. The genus origin and functional potential of the core genes were compared to those of all genes in the catalogue. The fraction of genes with an unknown genus origin is lower in the core genes compared to all genes in the catalogue (13% compared to 31%, respectively) (Table S8). The core genes were 20% from *Bacteroides* and 13% from *Clostridium* origin and these two genera were also the most common annotated genera in the full gene catalogue. At the functional level, a higher fraction of genes could be assigned to a gene in KEGG. A wide set of KEGG KOs had a higher annotation frequency to the core genes (Table S9). These functions include biosynthesis of secondary metabolites, amino acids and starch and sucrose metabolism. In summary, on average there is a shared common pool of genes but there is also a large number of genes in each individual that is shared with very few but are not completely unique.

## Discussion

The higher abundance of *Bacteroides* in the HMP and Chinese subjects compared to the European subjects can be due to differences in lifestyle, age, disease state, antibiotic use and diet. *Bacteroides* abundance has been associated with a diet high in animal protein, amino acids and saturated fats suggesting high meat consumption, *Prevotella* was found to be associated with high intake of carbohydrates and simple sugars [21]. It has also been observed that a diverse diet is associated with a diverse microbiota in an elderly population [19].

The gene catalogue presented here could be used for mapping of metagenomics sequence reads in future studies as it spans a large and diverse population. It clearly shows that there is a common core of genes across continents and populations although there are a many genes that are only found in few subjects. This indicates that more genes will be found when new subjects are studied but it is likely that these genes will have a very low abundance as the core genes found here have a high relative abundance. Possibly, some of the genes found in few individuals are transient genes whereas the core genes are more stable over time. The stable species of the microbiota has been found to be also the most abundant part by a 16S rRNA study using low error prone sequencing technology [22].

Differences in microbiota richness seen here between the European and Chinese and HMP studies can be due to a number of reasons. Antibiotic use, diet and other lifestyle effects are possible reasons for this difference. Also, methodological differences in sample collection and DNA extraction could influence sample richness and composition. The effect of antibiotics at subtherapeutic levels in mice is reduced diversity [23] and also in humans antibiotic use have been shown to have a major impact on the microbiota and reduced diversity [24]. The difference in diversity between the MetaHIT and HMP samples have also been seen in a previous study using phylogenetic marker genes [25]. In this study, this trend was seen both in species and gene richness and especially pronounced in the gene richness. It is likely that HMP samples which were sequenced to a greater depth have a higher proportion of their microbiome represented in the assemblies; this is also reflected in the large number of genes assembled from the HMP samples. However, the number of genes seen with a normalized number of reads is still substantially less than in the European samples.

In conclusion, we here present the MEDUSA pipeline, a tool for metagenomic data analysis with possibility for simultaneous taxonomic and gene annotation and handling of large data sets. We have applied this tool to perform the first comparison of four large studies from three continents and found a common species and gene core although the abundances of core components differ between populations. Furthermore, we provide a gene catalogue spanning over 11 million genes constructed from the different populations.

## Methods

### Implementation of the method

MEDUSA was implemented in python programming language and requires the numpy package (http://www.numpy.org/). MEDUSA makes use of standalone tools such as FASTX, bowtie2 [26] and GEM [27] that need to be callable from the Unix command line. The MEDUSA pipeline together with databases and results are available at http://www.metabolicatlas.com/medusa.

### Species catalogue construction

A non-redundant catalogue of genomes from prokaryotic species was constructed by using the results from grouping of prokaryotic genomes into species [14]. For each species, the longest of its member genomes was chosen as representative and the genome downloaded from NCBI Genbank. 8 genomes from the list were excluded as these records had been changed or retracted since the creation of the list of non-redundant species. All downloaded contigs were merged into a single fasta file and indexed by *gem-indexer*. The catalogue was annotated to NCBI taxonomy using the function *annotateToNCBITaxonomy* which creates an output file with taxonomy ids and taxnomomic names to each record in the reference catalogue.

### Gene catalogue construction

Four large metagenome studies were included in the construction of a global gut microbial gene catalogue. Assembled contigs were downloaded for the four studies [1], [2], [3], [4]. Genes were predicted on the contigs using Metagenemark [28]. Usearch [17] was used for constructing non-redundant sets of genes with 95% sequence identity and 90% coverage of the shorter sequence. This cutoff groups homologous genes from strains of the same species together but does generally not group more distantly related genes such as a protein family. A catalogue for each study was first constructed and then these were merged into a global catalogue.

### Data download and analysis

In this study, 782 human gut metagenomes were analyzed from four different studies, Sweden [3], MetaHIT [2], HMP [1] and China [4]. All samples were analyzed with the Illumina sequencing technology and a total of 40 billion reads were analyzed (Table S2). Some of the HMP subjects were sequenced on up to three occasions (Table S7). Each sequencing run was analyzed using the *streamAligner* function in MEDUSA and paired end reads were treated independently. Sequencing runs were merged into samples with the function *combineCounts* using a mapping file linking sequence runs to samples. The function *annotateCounts* was used on the gene count table to annotate counts to NCBI taxonomy and creating species and genus abundance tables.

### Gene counting and core analysis

Genes were considered present if two reads from the same sample aligned to it which is the same criteria used in by Qin et al. [2]. To normalize the sampling depth, the MEDUSA function *rarefy* was used to sample 11 million aligned reads from each subject.

In the analysis of core species and genes, HMP samples from visit 2 and 3 were removed to make sure that the core is defined on the individual basis and this reduced the number of samples from 782 to 719. The minimum relative abundance of a species to be counted as present in the core was 10^−4^ and the sensitivity to this cutoff for core species is shown in Figure S5.

### Enterotyping

Enterotypes were determined using the genus abundance with the methods suggested in http://enterotype.embl.de/ and in the paper by Arumugam et al [16], the analysis was performed in R using the package ade4.

### Data access

Data and software tools can be accessed through http://www.metabolicatlas.com/medusa.

## Supporting Information

Figure S1 Relative abundance of the 30 most abundant species in all 782 samples. Boxes denote the interquartile range (IQR) between the first and third quartiles and the line within denotes the median; whiskers denote the lowest and highest values within 1.5 times IQR from the first and third quartiles, respectively. Circles denote data points beyond the whiskers.
(PDF)

Figure S2 Boxplot of the 20 most abundant genera and their abundance by study. The definitions of boxplots are the same as in Figure S1.
(PDF)

Figure S3 Heatmap of relative abundance of the 30 most abundant species across 782 samples. Clustering was done using hierarchical clustering and complete linkage and Spearman correlation distance. Two clusters appear that are dominated by either Bacteroidetes species (*Bacteroides, Parabacteroides and Alistipes*) or Firmicutes species (*Faecalibacterium, Roseburia, Ruminococcus and Eubacterium*).
(PDF)

Figure S4 Heatmap of relative abundance of the 20 most abundant genera across 782 samples. Clustering was done using hierachical clustering and complete linkage and Spearman correlation distance.
(PDF)

Figure S5 Species core size as a function of the relative abundance cutoff shows that the pan size is more dependent on the cutoff than the core size.
(PDF)

Figure S6 Enterotype analysis of the samples. The recommended methods from http://enterotype.embl.de/ were used for the analysis. 73 genera with a mean abundance above 0.01% were used in the analysis. A) The clustering strength measured by Calinski-Harabasz index and the Silhouette index were calculated for a range of number of clusters. B) Between-class analysis using the R package ade4 for representing the genera abundance data together with the cluster identity as instrumental variable.
(PDF)

Figure S7 Abundance of three genera suggested being driver of each enterotype. Definitions of boxplots are the same as in Figure S1.
(PDF)

Figure S8 Histograms of abundance of three genera suggested to be drivers of enterotype separation. *Bacteroides* and *Ruminococcus* do not show a bimodal abundance distribution whereas *Prevotella* does.
(PDF)

Figure S9 Number of genes from each study. A) Number of genes predicted from contigs of each study. Genes from individual assemblies and global assemblies of unassembled reads are shown separately. B) Number of non-redundant genes in each study.
(PDF)

Figure S10 Relative abundance of genes grouped into how they are shared in the Venn diagram (Figure 3) and normalized to the number of genes in each section of the Venn diagram.
(PDF)

Figure S11 Number of gene in each sample using A) all data and B) data rarefied to 11 million aligned reads.
(PDF)

Table S1 List of species and genomes included in the species catalogue.
(XLSX)

Table S2 MEDUSA statistics for each sequencing run.
(XLSX)

Table S3 Pearson correlation between MEDUSA and Metaphlan genus abundance.
(XLSX)

Table S4 Identified core species in 50% and 90% of the individuals.
(XLSX)

Table S5 Enterotype distribution in each study.
(XLSX)

Table S6 Comparison of enterotypes assignment between this study and Arumugam et al [16].
(XLSX)

Table S7 Sample and repeated visit information.
(XLSX)

Table S8 Genus assignment of genes using the KEGG database. All refers to all 11 million genes in the gene catalogue while core refers to the core genes.
(XLSX)

Table S9 KO assignment of genes using the KEGG database. All refers to all 11 million genes in the gene catalogue while core refers to the core genes.
(XLSX)
