# Supplementary figures and images for: Metagenomic Data Utilization and Analysis (MEDUSA) and Construction of a Global Gut Microbial Gene Catalogue
Source: PLoS Comput Biol. 2014 Jul 10;10(7):e1003706. doi: 10.1371/journal.pcbi.1003706 (PMC4091689; doi:10.1371/journal.pcbi.1003706)

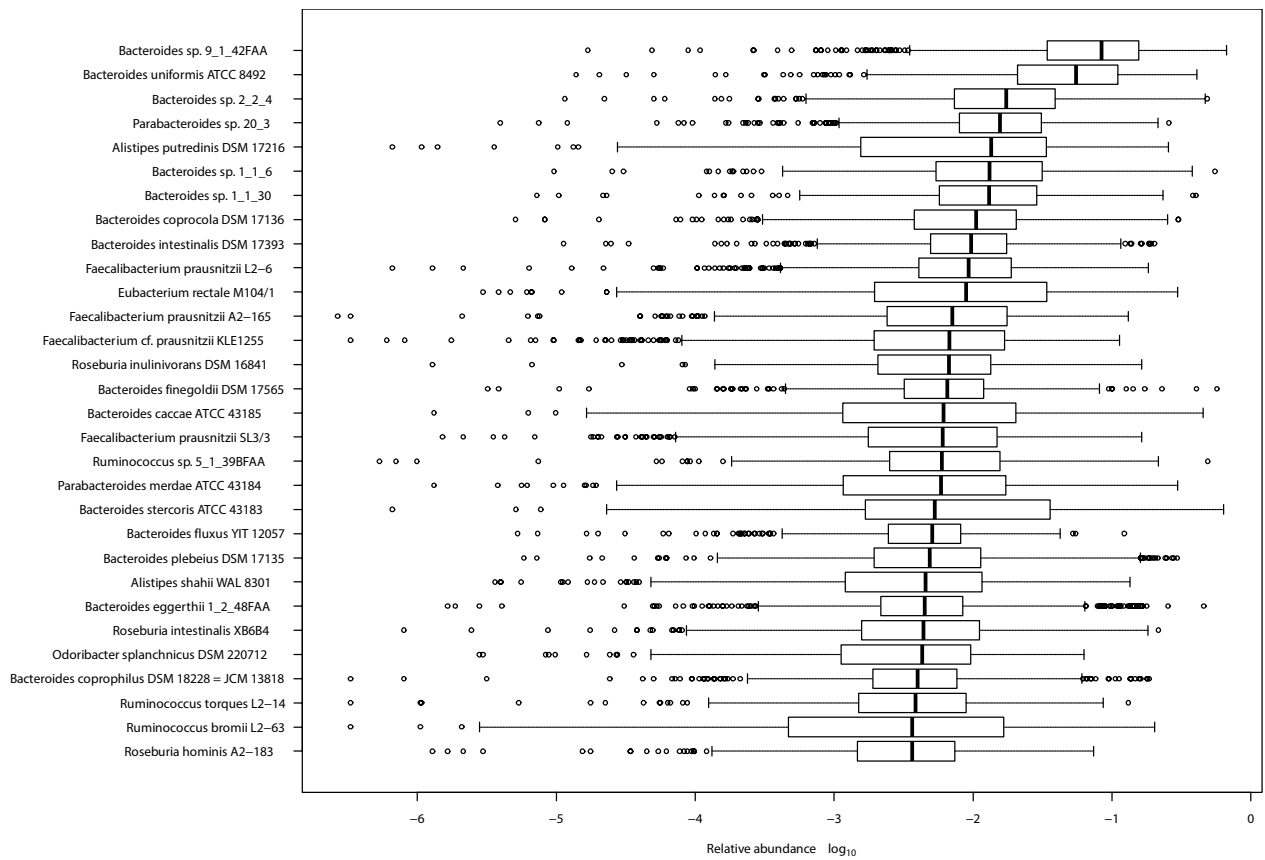

Supplement: Figure S1 — Relative abundance of the 30 most abundant species in all 782 samples. Boxes denote the interquartile range (IQR) between the first and third quartiles and the line within denotes the median; whiskers denote the lowest and highest values within 1.5 times IQR from the first and third quartiles, respectively. Circles denote data points beyond the whiskers. (PDF) [file pcbi.1003706.s001.pdf]

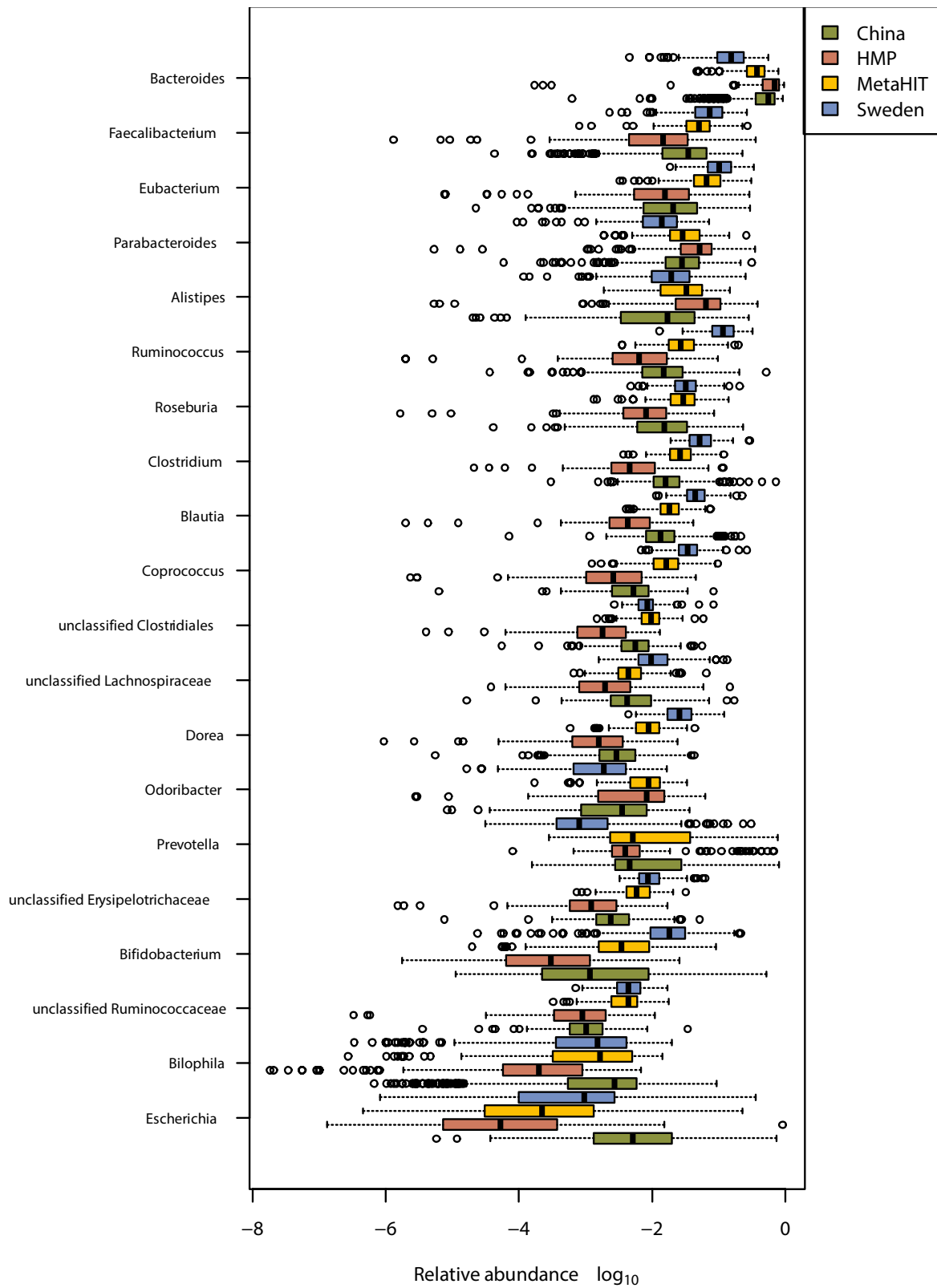

Supplement: Figure S2 — Boxplot of the 20 most abundant genera and their abundance by study. The definitions of boxplots are the same as in Figure S1. (PDF) [file pcbi.1003706.s002.pdf]

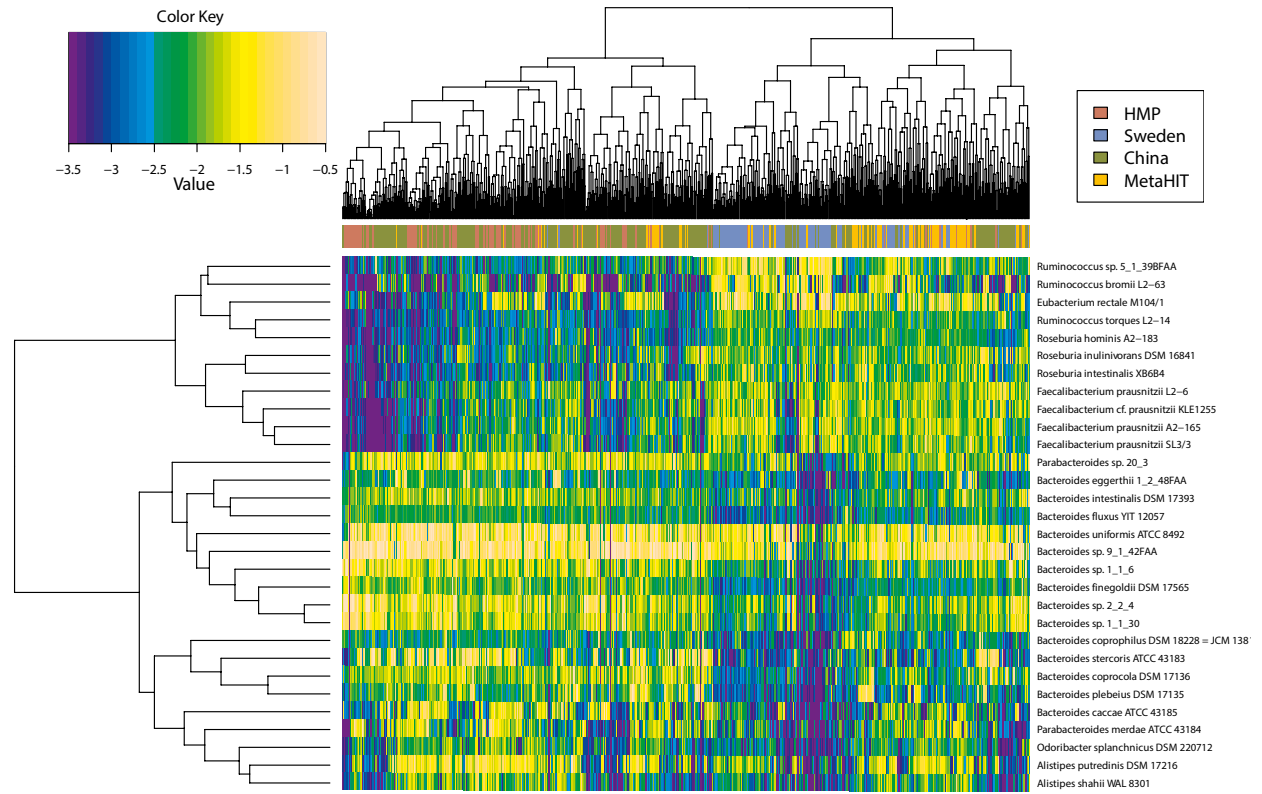

Supplement: Figure S3 — Heatmap of relative abundance of the 30 most abundant species across 782 samples. Clustering was done using hierarchical clustering and complete linkage and Spearman correlation distance. Two clusters appear that are dominated by either Bacteroidetes species (Bacteroides, Parabacteroides and Alistipes) or Firmicutes species (Faecalibacterium, Roseburia, Ruminococcus and Eubacterium). (PDF) [file pcbi.1003706.s003.pdf]

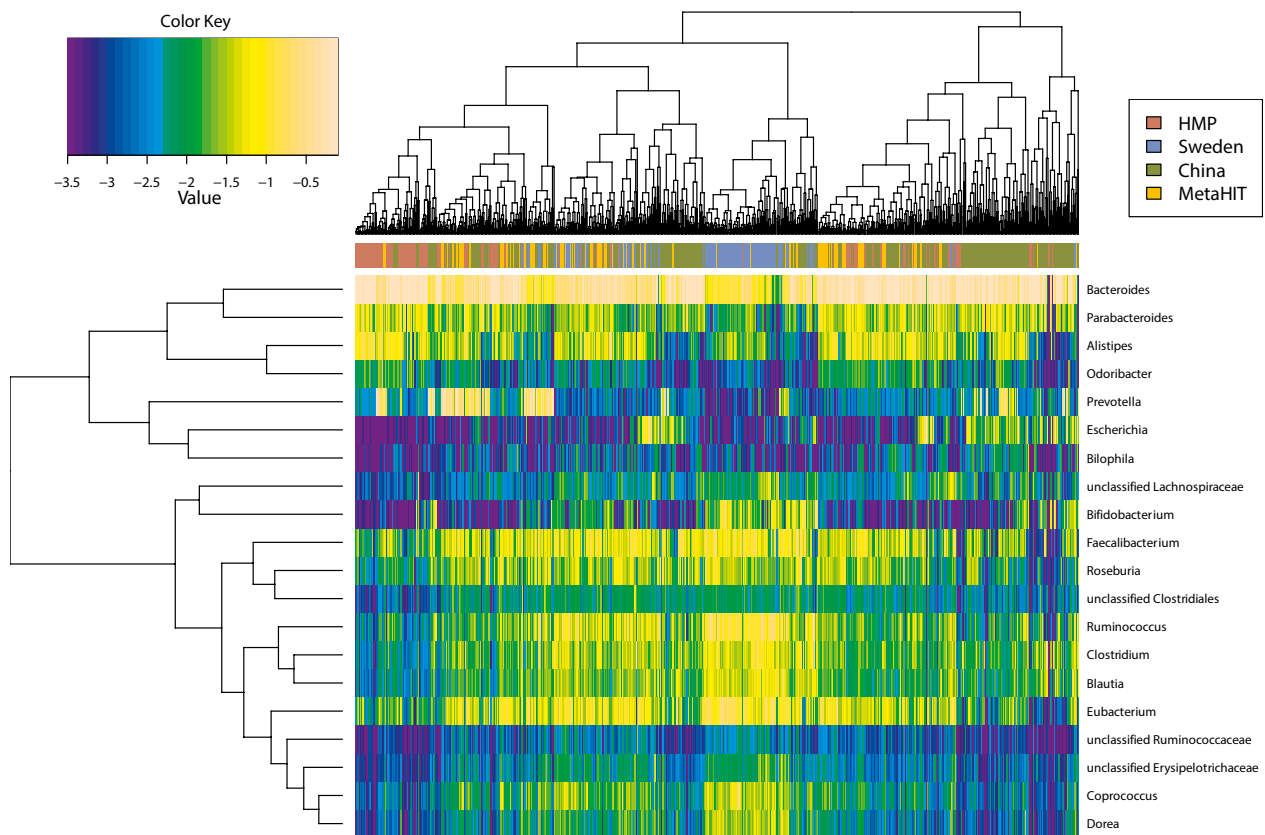

Supplement: Figure S4 — Heatmap of relative abundance of the 20 most abundant genera across 782 samples. Clustering was done using hierachical clustering and complete linkage and Spearman correlation distance. (PDF) [file pcbi.1003706.s004.pdf]

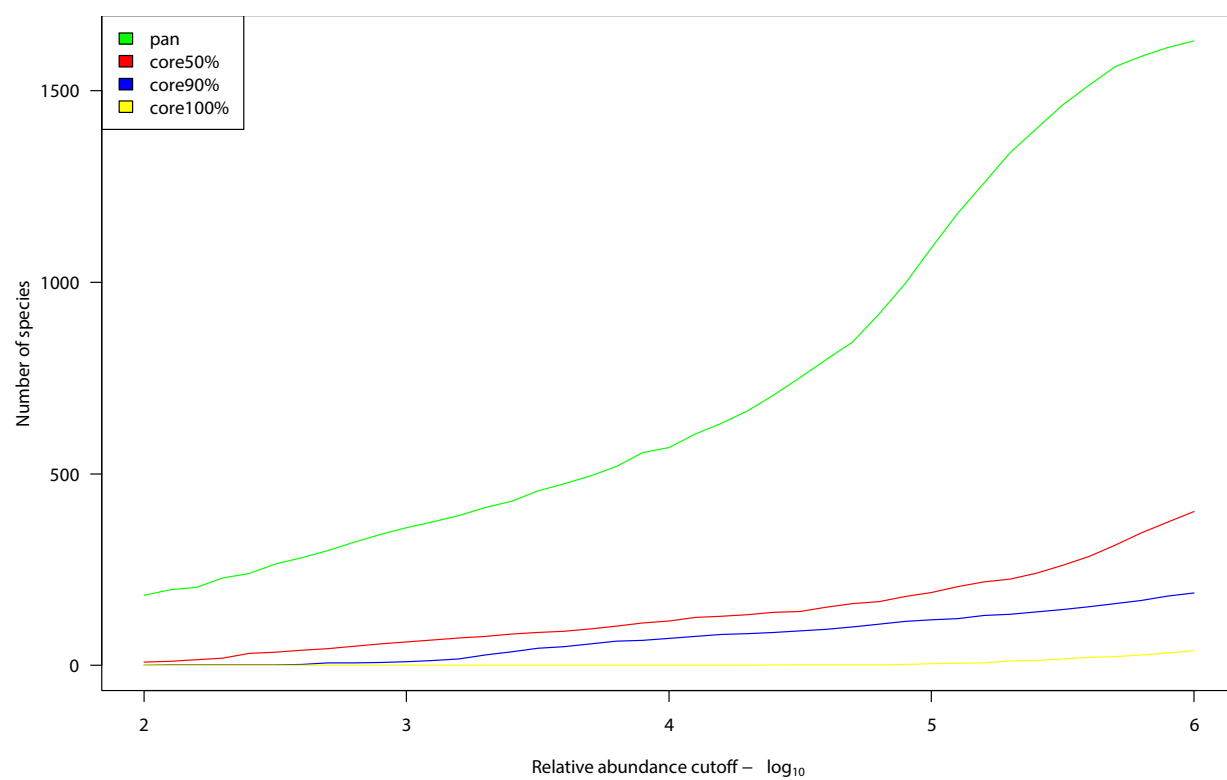

Supplement: Figure S5 — Species core size as a function of the relative abundance cutoff shows that the pan size is more dependent on the cutoff than the core size. (PDF) [file pcbi.1003706.s005.pdf]

**A**

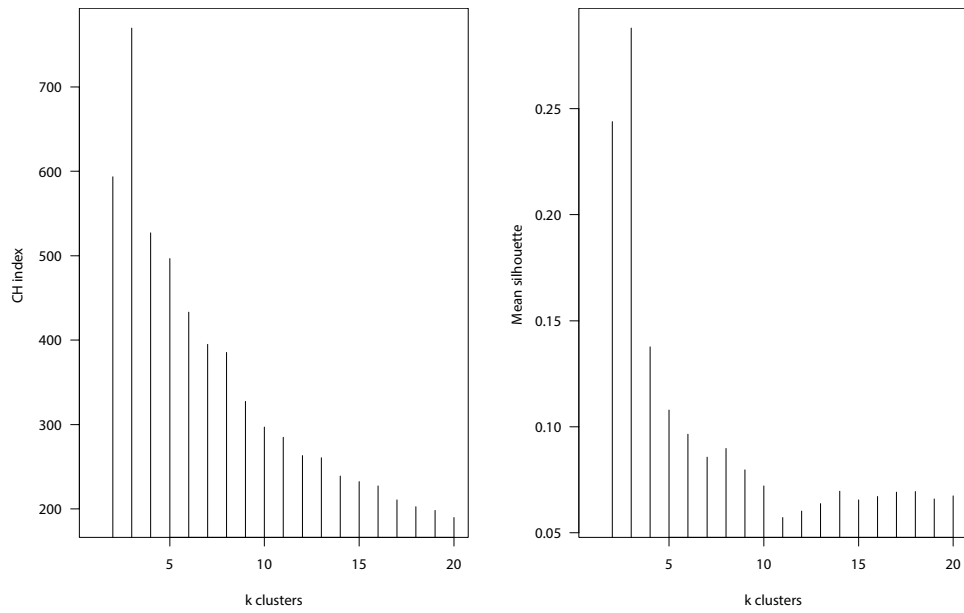

**B**

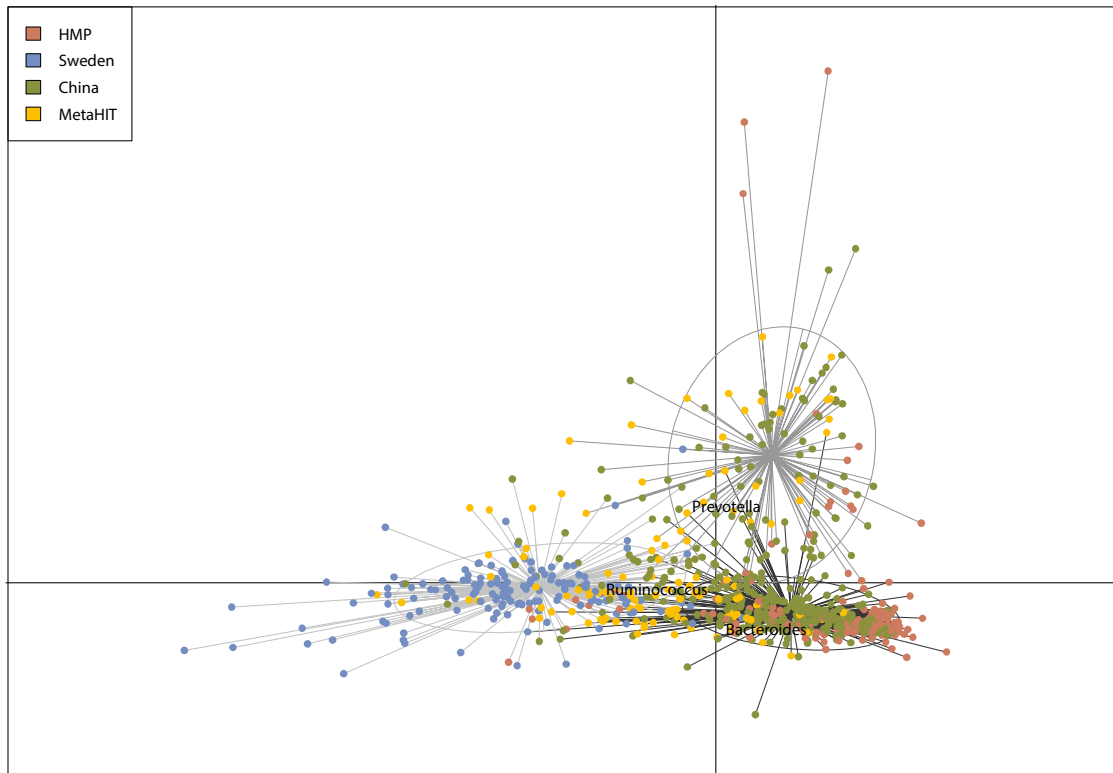

Supplement: Figure S6 — Enterotype analysis of the samples. The recommended methods from http://enterotype.embl.de/ were used for the analysis. 73 genera with a mean abundance above 0.01% were used in the analysis. A) The clustering strength measured by Calinski-Harabasz index and the Silhouette index were calculated for a range of number of clusters. B) Between-class analysis using the R package ade4 for representing the genera abundance data together with the cluster identity as instrumental variable. (PDF) [file pcbi.1003706.s006.pdf]

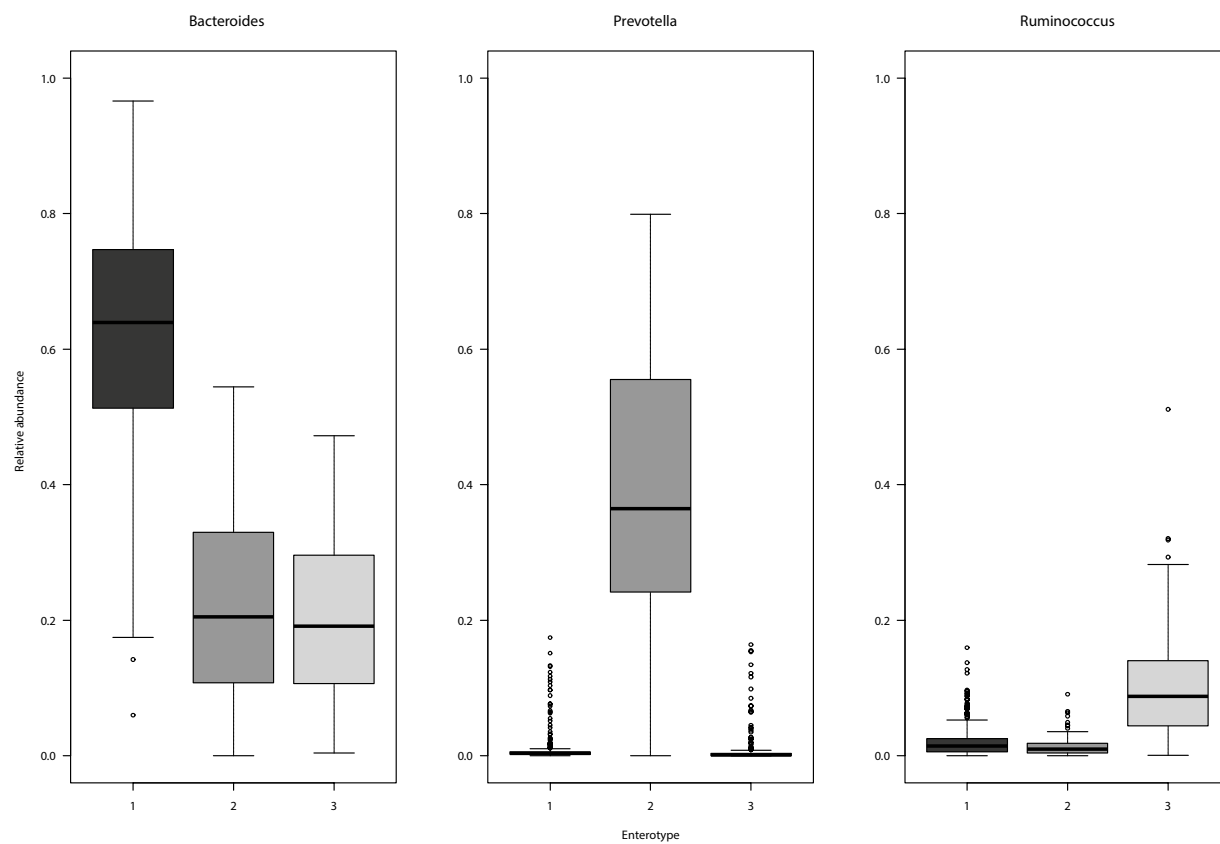

Supplement: Figure S7 — Abundance of three genera suggested being driver of each enterotype. Definitions of boxplots are the same as in Figure S1. (PDF) [file pcbi.1003706.s007.pdf]

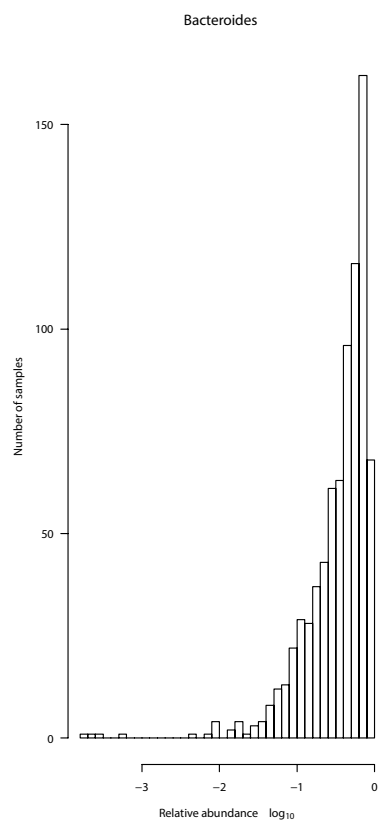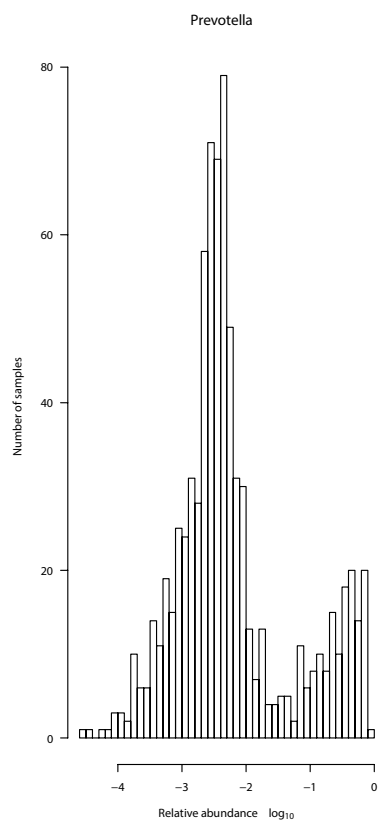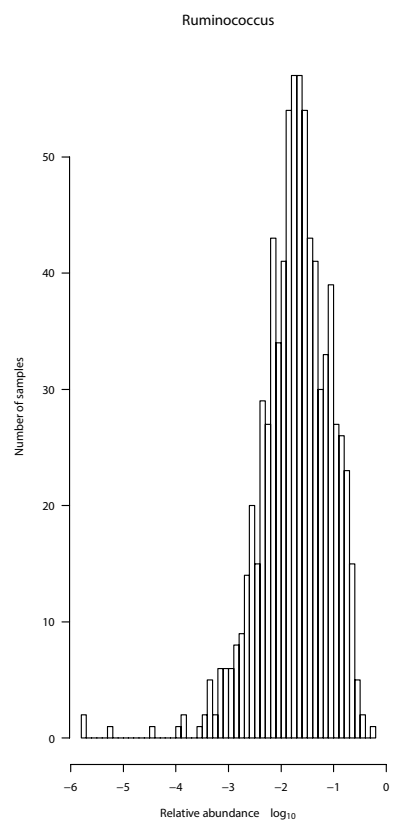

Supplement: Figure S8 — Histograms of abundance of three genera suggested to be drivers of enterotype separation. Bacteroides and Ruminococcus do not show a bimodal abundance distribution whereas Prevotella does. (PDF) [file pcbi.1003706.s008.pdf]

A

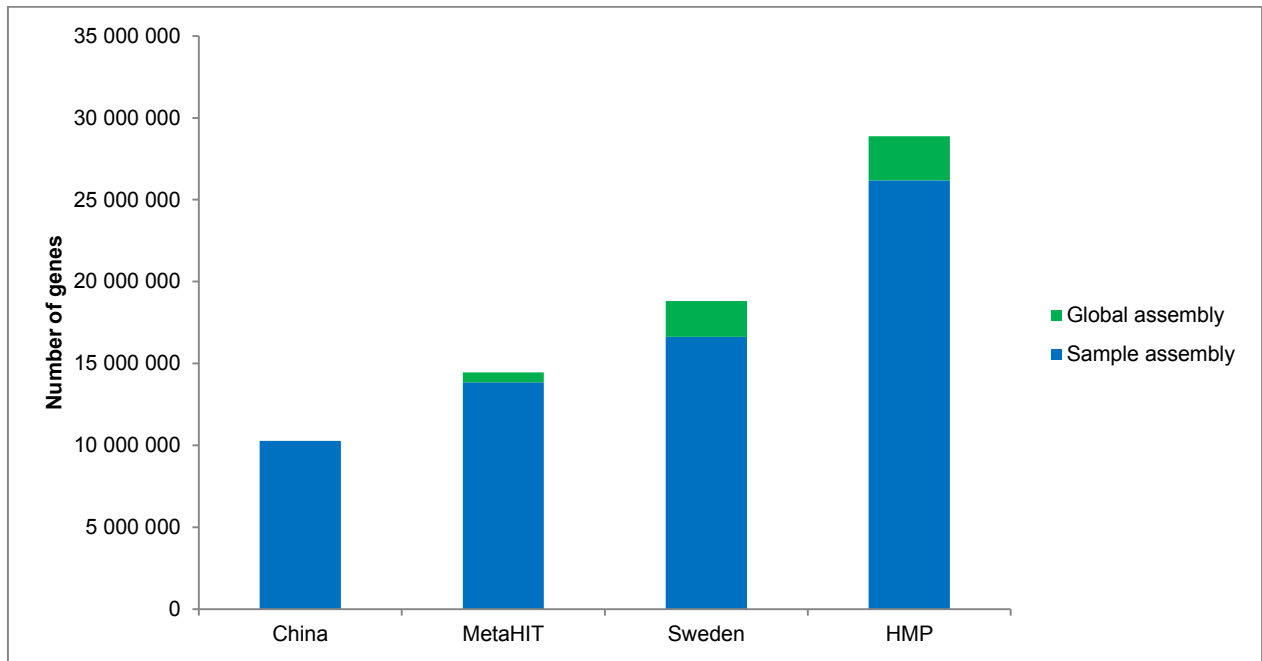

B

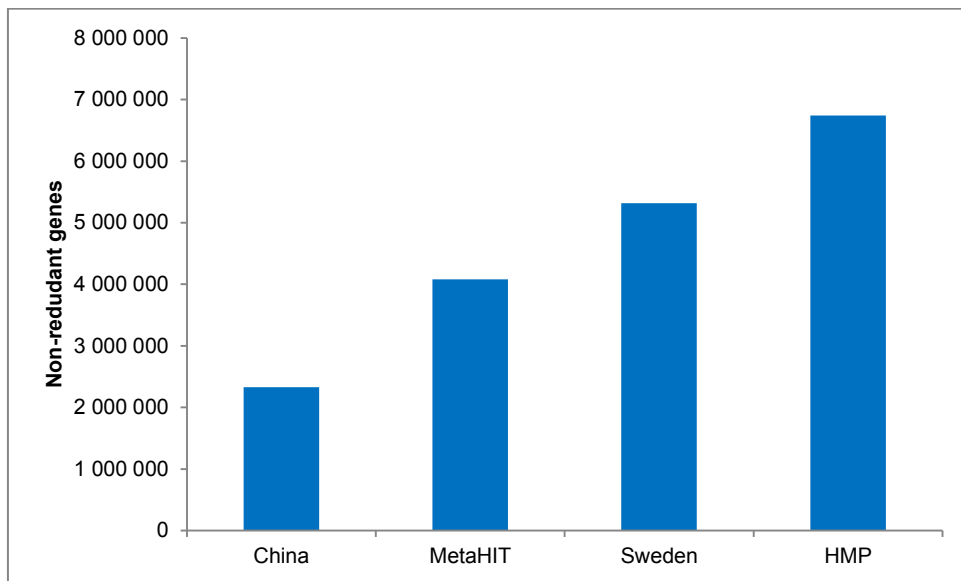

Supplement: Figure S9 — Number of genes from each study. A) Number of genes predicted from contigs of each study. Genes from individual assemblies and global assemblies of unassembled reads are shown separately. B) Number of non-redundant genes in each study. (PDF) [file pcbi.1003706.s009.pdf]

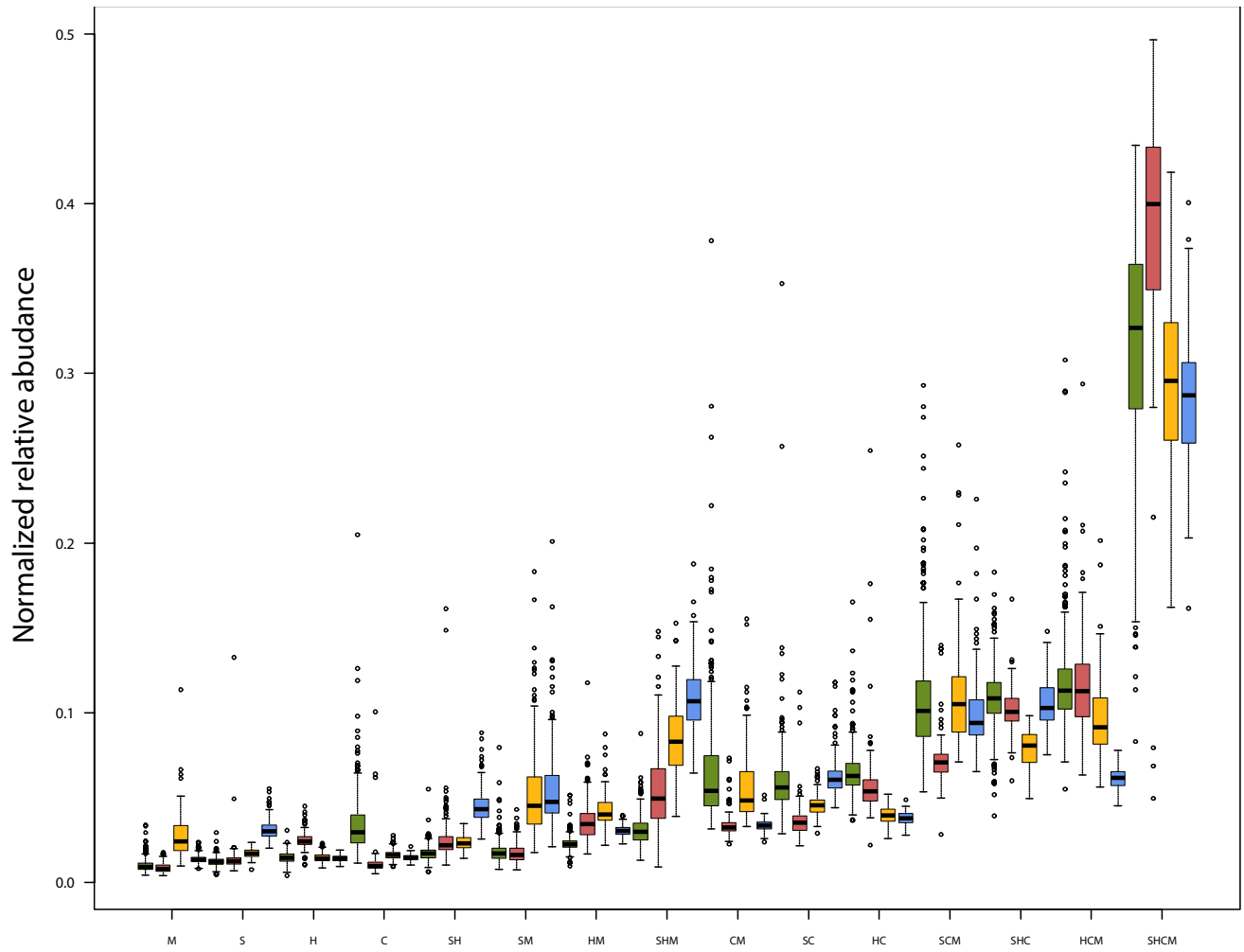

Supplement: Figure S10 — Relative abundance of genes grouped into how they are shared in the Venn diagram (Figure 3) and normalized to the number of genes in each section of the Venn diagram. (PDF) [file pcbi.1003706.s010.pdf]

A

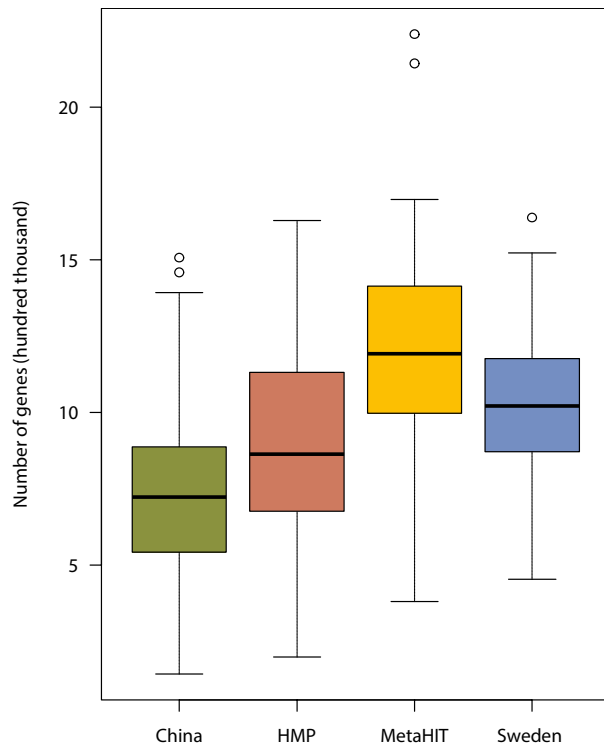

B

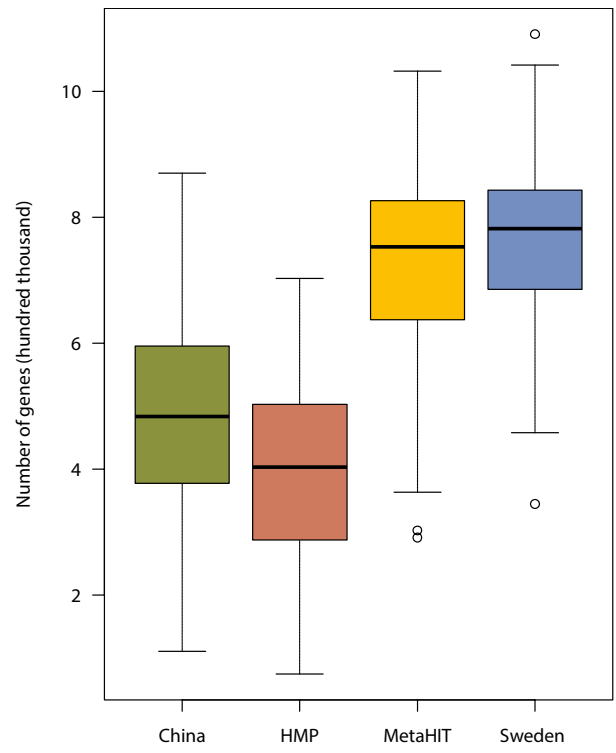

Supplement: Figure S11 — Number of gene in each sample using A) all data and B) data rarefied to 11 million aligned reads. (PDF) [file pcbi.1003706.s011.pdf]
